# Gastrointestinal Bleeding due to Pancreatic Disease-Related Portal Hypertension

**DOI:** 10.1155/2020/3825186

**Published:** 2020-03-27

**Authors:** Kexin Zheng, Xiaozhong Guo, Ji Feng, Zhaohui Bai, Xiaodong Shao, Fangfang Yi, Yongguo Zhang, Rui Zhang, Han Liu, Fernando Gomes Romeiro, Xingshun Qi

**Affiliations:** ^1^Department of Gastroenterology, General Hospital of Northern Theater Command (Formerly General Hospital of Shenyang Military Area), Shenyang 110840, China; ^2^Postgraduate College, Jinzhou Medical University, Jinzhou 121000, China; ^3^Postgraduate College, Shenyang Pharmaceutical University, Shenyang 110016, China; ^4^Postgraduate College, Dalian Medical University, Dalian 116044, China; ^5^Departamento de Clínica Médica, Faculdade de Medicina de Botucatu, Universidade Estadual Paulista (UNESP), Botucatu, SP, Brazil

## Abstract

**Background and Aims:**

Left-sided portal hypertension (LSPH) is a rare type of portal hypertension, which occurs due to obstruction, stenosis, or thrombosis within the splenic vein. Pancreatic diseases are the most common etiology of LSPH. This study is aimed at reporting our experiences and discussing the presentation, management, and prognosis of LSPH secondary to pancreatic diseases. *Patients and Methods*. We retrospectively reviewed five patients who were diagnosed with LSPH secondary to pancreatic diseases at our department. We collected the demographic information, history, comorbidities, clinical presentations, laboratory tests, esophagogastroduodenoscopy (EGD), images, and outcome data.

**Results:**

Three elderly patients (>60 years old) were diagnosed with pancreatic cancer, of whom one underwent laparoscopic radical distal pancreatectomy and splenectomy, one received chemotherapy, and another one chose conservative management due to multiple systemic metastases. Two younger patients (<40 years old) were diagnosed with acute recurrent pancreatitis and chronic pancreatitis. Four of these five included patients presented with hematemesis and/or melena at our admission. All patients had gastric varices, and one of them also had esophageal varices. One elderly patient with metastatic pancreatic cancer underwent endoscopic variceal treatment as a rescue therapy but finally died of refractory gastrointestinal (GI) bleeding; another younger patient with chronic pancreatitis died of massive GI bleeding; and the remaining three patients survived at their last follow-up.

**Conclusions:**

LSPH should be seriously taken into consideration in patients with pancreatic diseases who develop upper GI bleeding. Clinicians should individualize the treatment strategy of LSPH according to the patients' clinical conditions and nature of pancreatic diseases.

## 1. Introduction

Left-sided portal hypertension (LSPH) refers to a rare type of extrahepatic portal hypertension secondary to the obstruction or stenosis of splenic vein [1]. In cases with LSPH, the splenic blood outflow diverts to low-pressure collaterals, such as short gastric, gastric coronary, and gastroepiploic veins. Most of LSPH patients are asymptomatic but can present with gastrointestinal (GI) bleeding from gastric varices with or without esophageal varices [1, 2]. The most common etiologies of LSPH are pancreatic diseases including chronic pancreatitis, pancreatic pseudocyst, and pancreatic neoplasms. Other etiologies include iatrogenic injury to the splenic vein during surgical procedures and infiltrating colonic cancer [3, 4]. Resection of the obstructing or infiltrating primary lesion is considered as the radical choice of therapy [5]. However, most patients with advanced malignancy are not eligible for surgical procedures. Other treatment modalities include splenectomy, splenic arterial embolization, endovascular recanalization, and endoscopic variceal treatment [2, 6]. Notably, endoscopic variceal treatment is considered as a rescue therapy for massive acute GI bleeding but is associated with a high rate of recurrence [6–8]. Till now, there is no guideline or consensus regarding epidemiology, diagnosis, and treatment of LSPH because of too limited evidence. It remains a challenge for clinicians to make a rapid and appropriate decision on the diagnosis and treatment of LSPH. Therefore, it is very important to share clinical experiences which can provoke the clinicians' consciousness of LSPH and its complications. Herein, we report a case series of five patients with LSPH secondary to pancreatic diseases who underwent endoscopic and surgical management.

## 2. Patients and Methods

We retrospectively reviewed the medical records of five patients who were diagnosed with LSPH secondary to pancreatic diseases via abdominal computed tomography (CT) or magnetic resonance (MR) and esophagogastroduodenoscopy (EGD) and treated at our department between October 2018 and August 2019. We collected the demographic information, clinical presentations, comorbidities, laboratory tests, EGD, images, and outcome data. Their detailed information was as follows.

### 2.1. Case 1 (TL)

**Figure 1:**
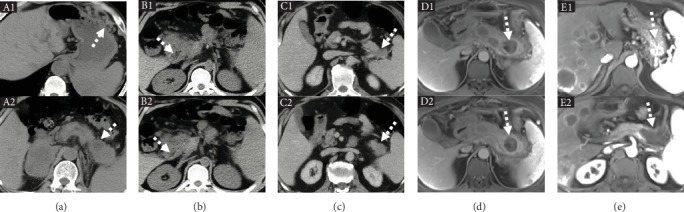
Images regarding pancreatic lesions in five LPSH patients. (a) Plain abdominal CT of case 1 (TL) demonstrating multiple distorted soft issue mass densities in the left upper abdomen (A1, white dotted arrow) and a cystic lesion in the pancreatic tail (A2, white dotted arrow). (b) Plain abdominal CT of case 2 (YC) demonstrating a distorted pancreatic head with local hypodensity (B1 and B2, white dotted arrows). (c) Contrast-enhanced abdominal CT of case 3 (WZ) demonstrating a mass in the pancreatic tail (C1 and C2, white dotted arrows). (d) Contrast-enhanced abdominal MR of case 4 (JH) demonstrating acute hemorrhagic necrotizing pancreatitis with pancreatic pseudocyst (D1 and D2, white dotted arrows). (e) Contrast-enhanced abdominal MR of case 5 (HW) demonstrating multiple gastric and splenic varices (E1, white dotted arrow) and pancreatic cystadenocarcinoma in the pancreatic tail (E2, white dotted arrow).

**Figure 2:**
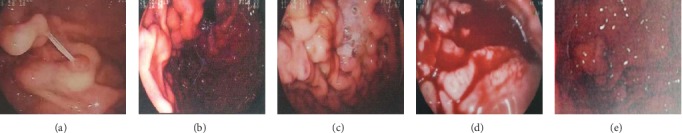
EGD regarding varices in five LPSH patients. (a) EGD of case 1 (TL) demonstrating multiple gastric varices with a red color sign. (b) EGD of case 2 (YC) demonstrating multiple varices in the stomach with a red color sign. (c) EGD of case 3 (WZ) demonstrating multiple gastric varices with a red color sign and bloodstain. (d) EGD of case 4 (JH) demonstrating multiple gastric varices with bloodstain. (e) EGD of case 5 (HW) demonstrating multiple gastric varices.

On October 26, 2018, a 68-year-old male was admitted to our department with complaints of melena for six hours. The patient had a past medical history of left renal clear cell cancer with metastasis to the lung, thyroid, and bone. He had undergone left nephrectomy, left upper lobectomy, thyroidectomy, and left upper limb osteotomy in 2006, 2012, 2015, and 2017, respectively. Before this admission, he had undergone endoscopic glue adhesive injection for isolated gastric variceal bleeding at our institution. On August 30, 2018, a plain abdominal CT scan demonstrated abnormal pancreatic head, a cystic lesion in the pancreatic tail, and multiple distorted soft tissue densities in the left upper abdomen (Figure 1(a)). The patient did not undergo contrast-enhanced CT scans because of his severe allergy to the contrast media. A diagnosis of metastatic pancreatic cancer was considered. Patient's GI bleeding did not respond to the initial treatment with conservative pharmacological therapies. Our surgeons did not consider surgery because he had a previous history of multiple surgery and presented with extreme weakness and multiple metastasis of renal cancer. The patient also refused to undergo any further surgical procedures. Then, he underwent EGD which demonstrated multiple varices with a red color sign in the fundus and body of the stomach (Figure 2(a)). He underwent endoscopic glue adhesive injection for gastric variceal bleeding. However, GI bleeding recurred several days after this endoscopic procedure. Interventional endovascular procedures could not be considered because of his allergy to the contrast media. Surgical procedures were also not undertaken because of his multimorbid condition. Ultimately, he died of uncontrolled GI bleeding, hemorrhagic shock, and multiple organ failure on January 16, 2019.

### 2.2. Case 2 (YC)

On February 24, 2019, a 33-year-old male was admitted to our department with complaints of melena for four days and hematemesis for two days. The patient had past histories of three episodes of acute pancreatitis and type 2 diabetes mellitus for 3 years. A plain abdominal CT scan demonstrated a distorted pancreatic head with local hypodensity and splenomegaly (Figure 1(b)). He was diagnosed with chronic pancreatitis. The patient was started on intravenous proton pump inhibitor (PPI) and octreotide. He underwent EGD which demonstrated multiple varices with a red color sign in the fundus and body of the stomach as well as in the esophagus (Figure 2(b)). Our endoscopist decided not to perform endoscopic variceal treatment due to extensive esophagogastric varices and its limited effectiveness in LSPH. He did not develop melena or hematemesis again during this hospitalization. Then, the patient refused further treatment and was discharged on February 27, 2019. At a telephone follow-up, he died of massive GI bleeding on September 24, 2019.

### 2.3. Case 3 (WZ)

On April 8, 2019, a 67-year-old male was admitted to our department with complaints of weakness and chest distress for twenty days. On April 18, 2019, a contrast-enhanced abdominal CT scan demonstrated a mass in the pancreatic tail and mild splenomegaly (Figure 1(c)). An abdominal vascular ultrasound demonstrated that the splenic vein diameter was 5.8 mm, the splenic artery diameter was 4.8 mm, and the splenic artery flow velocity was increased to 2.98 m/s. He underwent EGD which demonstrated multiple varices with a red color sign and bloodstain in the fundus and body of the stomach (Figure 2(c)). Our endoscopist decided not to perform endoscopic variceal treatment. Then, the patient underwent laparoscopic radical distal pancreatectomy and splenectomy at another hospital. The histopathological analysis demonstrated moderately differentiated pancreatic ductal adenocarcinoma. At a telephone follow-up on December 6, 2019, he was stable without any complaint of hematemesis or melena. A repeat EGD failed to show the presence of gastric varices, thus suggesting their disappearance after the surgery.

### 2.4. Case 4 (JH)

On May 17, 2019, a 36-year-old male was admitted to our department with complaints of hematemesis and melena for two days and 10 kg weight loss during the past month. He had a history of recurrent acute pancreatitis for several times during the past decade. His last episode of acute pancreatitis occurred one month ago. A plain abdominal CT scan demonstrated acute pancreatitis with bilateral pleural effusion and mild ascites. The patient was started on PPI and somatostatin drips. On May 19, 2019, a contrast-enhanced abdominal MR scan demonstrated acute hemorrhagic necrotizing pancreatitis with pancreatic pseudocyst, bilateral pleural effusion, and mild ascites (Figure 1(d)). He underwent EGD which demonstrated multiple varices with bloodstain in the gastric fundus (Figure 2(d)). Our endoscopist did not perform endoscopic variceal treatment. On May 27, 2019, a repeated contrast-enhanced abdominal CT scan demonstrated pancreatitis, pancreatic pseudocyst, and multiple enlarged lymph nodes in the abdominal cavity and retroperitoneum. Ultimately, the patient's clinical condition improved and he was discharged. At a telephone follow-up on December 6, 2019, the patient was relatively stable without any complaints of hematemesis or melena. However, he did not undergo follow-up abdominal CT or EGD since he was asymptomatic.

### 2.5. Case 5 (HW)

On August 16, 2019, a 64-year-old male was admitted to our department due to sudden onset of hematemesis for one hour. A contrast-enhanced abdominal MR scan demonstrated pancreatic cystadenocarcinoma in the pancreatic tail and body with multiple intrahepatic metastases and splenomegaly (Figure 1(e)). He underwent EGD which demonstrated multiple varices in the gastric fundus with active bleeding (Figure 2(e)). Our endoscopist just performed endoscopic spraying fluid film, but not endoscopic variceal treatment. The patient received chemotherapy, but his cancer continued to progress with an increased number of metastatic lesions and recurrence of GI bleeding. Then, he was transferred to palliative and supportive care. He was discharged on November 18, 2019.

## 3. Results

A total of five patients with LSPH secondary to pancreatic diseases were included. All of them were male. Three elderly patients with an age of 64-68 years old had pancreatic cancer, of whom one underwent laparoscopic radical distal pancreatectomy and splenectomy, one underwent chemotherapy, and another one received conservative therapy alone. Two younger patients with an age of 33-36 years old were diagnosed with pancreatitis, of whom one had chronic pancreatitis and another had recurrent acute pancreatitis.

Four of the five patients presented with chief complaints of hematemesis and/or melena to our department. All of them had gastric varices on EGD. One of them also had esophageal varices, but he did not have other stigmata of liver cirrhosis.

Major laboratory tests and imaging characteristics are summarized in Table 1. Four of the five patients had anemia. Only one patient had elevated levels of alanine aminotransaminase, aspartate aminotransferase, and alkaline phosphatase, and two patients had an elevated level of *γ*-glutamyl transpeptidase.

One patient with metastatic pancreatic cancer underwent endoscopic variceal treatment as a rescue therapy but finally died of uncontrolled GI bleeding. Another patient with chronic pancreatitis did not undergo endoscopic variceal treatment at our department and then was discharged, but he finally died of recurrent massive GI bleeding at his local hospital. The remaining three patients did not undergo endoscopic variceal treatment at our department but survived at their last follow-up.

## 4. Discussion

The pathophysiology and mechanisms of portal hypertension, clinical presentations, treatment, and outcomes vary extremely between patients with pancreatic diseases with normal liver architecture and function and cirrhotic patients [9, 10]. Patients with pancreatic diseases can develop LSPH either due to intrinsic stenosis, or thrombosis of splenic vein because of inflammation and hypercoagulability [11, 12], or obstruction due to extrinsic compression. Liver architecture distortion and increased intrahepatic portal inflow resistance are more important for development of portal hypertension in liver cirrhosis [13]. Isolated gastric varices are the most important source of GI bleeding in the patients with LSPH, whereas esophageal varices with or without gastric varices are more common in cirrhotic patients [14].

For patients with pancreatic diseases and LSPH, the treatment selection is often based on the patients' clinical condition and nature of pancreatic disease. The distal pancreatectomy and splenectomy is a relatively safe and effective choice to completely alleviate portal hypertension and varices in patients with pancreatic cancer [5, 15]. However, it can be difficult and challenging to perform complex surgical procedures in multimorbid patients with metastatic pancreatic cancer. Splenic artery embolization can also be considered as an alternative choice of treatment [16]. However, portosystemic shunt should not be considered in LSPH because portal pressure and liver function are often normal in these patients [1]. Endoscopic variceal treatment is only considered in patients with life-threatening bleeding from LSPH. Indeed, the efficacy of endoscopic variceal treatment is often questioned and may increase the risk of recurrent bleeding because it can increase the pressure of collaterals after blocking the outflow tract of splenic blood [17]. Sometimes, an effective treatment of pancreatic pseudocyst is useful for improving LSPH [18].

We reviewed the literature for the case reports [19–26] and case series [16, 27–30] about prognosis of LSPH secondary to the pancreatic diseases (Tables 2 and 3). Somehow, the prognosis of LSPH mainly depends on the nature of primary pancreatic disease and severity of refractory GI bleeding [7, 20]. It is well known that the outcomes of pancreatic cancer are worse than those of pancreatitis. The prognosis of pancreatic cancer is dismal with a 5-year survival rate of 6% [31]. The outcomes of pancreatic cancer also depend on the tumor staging at the time of diagnosis [32]. Only 20% of patients with pancreatic cancer have a chance to undergo surgical procedures [33]. By contrast, the prognosis of pancreatitis is better and often depends on its severity and complications [34, 35]. The prognosis of severe acute pancreatitis is significantly worse than that of moderately severe acute pancreatitis [36]. The complications, which include pancreatic pseudocysts, stenosis of the common bile duct, pleural effusion, and ascites, severely implicate the prognosis of pancreatitis [37]. On the other hand, massive and recurrent GI bleeding is also a major cause of death, especially in patients who did not receive any effective treatment for splenic vein obstruction.

The limitation of our study is very obvious that it is a small case series and is lacking of control groups. Therefore, we cannot get a definite conclusion regarding the preferred choice of treatment for LSPH secondary to pancreatic diseases. In the future, comparative studies will be helpful to explore the difference in outcomes among the LSPH patients who had comparable baseline characteristics but received different treatments.

In conclusion, LSPH secondary to the pancreatic diseases is rare in the clinical practice. We have reported a case series of five patients with pancreatic diseases who presented to us with GI bleeding related to LSPH. LSPH should be seriously taken into consideration in patients with pancreatic diseases who develop upper GI bleeding. Clinicians should individualize the treatment strategy of LSPH according to the patients' clinical conditions and nature of pancreatic diseases. Further prospective studies are needed to systematically explore the epidemiology, anatomy, pathophysiology, diagnosis, treatment, and prognosis of LSPH in the pancreatic diseases.

## Figures and Tables

**Table 1:** Characteristics of the patients with left-sided portal hypertension.

Variables	1 (TL)	2 (YC)	3 (WZ)	4 (JH)	5 (HW)
Gender	Male	Male	Male	Male	Male
Age (years)	68	33	67	36	64
Pancreatic diseases	Pancreatic cancer	Pancreatitis	Pancreatic cancer	Pancreatitis	Pancreatic cancer
Locations of the pancreatic diseases	Head, body, and tail	Head	Tail	Body and tail	Body and tail
Clinical presentation	Melena	Hematemesis and melena	Blood in stomach	Hematemesis and melena	Hematemesis
Gastrointestinal bleeding at admission	Yes	Yes	Yes	Yes	Yes
Comorbidities	Left renal clear cell cancer Left upper lung lobe metastatic cancer Thyroid metastatic cancer Left upper limb bone metastatic cancer	Recurrent pancreatitis Diabetes	Hypertension	Recurrent pancreatitis Coronary heart disease Hypertension	Lacunar infarction
EGD	Gastric varices	Esophagogastric varices	Gastric varices	Gastric varices	Gastric varices
Laboratory tests at admission					
WBC (10^9^/L)	3.4	4.9	5.1	8.6	4.9
GR (%)	63.4	75.8	66.8	66.5	79.2
RBC (10^12^/L)	2.63	4.27	3.05	4.00	4.01
HB (g/L)	74	111	76	108	125
PLT (10^9^/L)	78	71	209	205	78
TBIL (*μ*mol/L)	15.4	15.1	7.7	27.1	17.4
DBIL (*μ*mol/L)	9.2	3.7	2.2	7.8	4.4
ALT (U/L)	116.02	14.55	12.59	8.09	23.33
AST (U/L)	109.59	15.4	17.14	9.75	30.92
AKP (U/L)	361.51	60.66	88.37	81.02	96.56
GGT (U/L)	199.51	23.41	18.4	32.31	124
AMY (U/L)	79.00	52.00	NA	92.00	145.00
LIPA (U/L)	457.0	45.0	NA	394.0	52.0
Types of treatment	Endoscopic variceal treatment Pharmacological treatment	Pharmacological treatment	Distal pancreatectomy and splenectomy Pharmacological treatment	Pharmacological treatment	Endoscopic spraying fluid film Chemotherapy Pharmacological treatment
Outcomes	Died	Died	Survived	Survived	Survived

Abbreviations: EGD: esophagogastroduodenoscopy; WBC: white blood cell; GR%: neutrophil percentage; RBC: red blood cell; HB: hemoglobin concentration; PLT: platelet; TBIL: total bilirubin; DBIL: direct bilirubin; ALT: alanine aminotransaminase; AST: aspartate aminotransferase; AKP: alkaline phosphatase; GGT: *γ*-glutamyl transpeptidase: NA: not available; AMY: amylase; LIPA: lipase. The reference range of laboratory tests: WBC: 3.5‐9.5 × 10^9^/L; GR%: 40-75%; RBC: 4.3‐5.8 × 10^12^/L; HB: 115‐175 g/L; PLT: 125‐350 × 10^9^/L; TBIL: 5.1-20.0 *μ*mol/L; DBIL: 0-6.8 *μ*mol/L; ALT: 7-50 U/L; AST: 13-40 U/L; AKP: 13-150 U/L; GGT: 7-60 U/L; AMY: 30-110 U/L; LIPA: 23-300 U/L.

**Table 2:** Literature review of case reports depicting the prognosis of patients with left-sided portal hypertension.

References	Country	Age (years)	Gender	Clinical presentations at admission	Primary pancreatic diseases	Location of varices	Interventions	Follow-up period	Outcome
Singhal et al. [19]	India	63	Female	Hematemesis and melena	Pancreatic cystadenoma	Gastric varices	Distal pancreatectomy and splenectomy	5 years	Survived
Thompson et al. [20]	UK	57	Female	Upper gastrointestinal bleeding	Pancreatic pseudocyst	Gastric varices	Cystotomy and splenectomy	NA	Survived
		53	Male	Melena	Pancreatic somatostatinoma	Gastric varices	NA		Survived
		42	Female	Upper gastrointestinal bleeding	Metastatic pancreatic cancer	Gastric varices	Laparotomy of gastric variceal ligation		NA
Ito et al. [21]	Japan	68	Male	No presentation	Pancreatic serous cystadenoma	Gastric varices	Distal pancreatectomy plus splenectomy	NA	Survived
Franzoni et al. [22]	Brazil	19	Female	Hematemesis	Pancreatic hemangioma	Esophagogastric varices	Endoscopic band ligation and *β*-blocker treatment before childbirth Distal pancreatectomy and splenectomy after childbirth	7 years	Survived
Li et al. [23]	China	34	Female	Acute epigastric pain, hematemesis, and melena	Acute pancreatitis	Gastric and perisplenic varices	Splenic arterial embolization	5 months	Survived
El Kininy et al. [24]	Ireland	38	Male	Epigastric pain and vomiting	Acute pancreatitis	Splenic varices	Laparotomy, interventional drainages, and splenic vein stent	11.5 months	Survived
Canbak et. al. [25]	Turkey	26	Male	Abdominal pain	Pancreatic hydatid cyst	Splenic hilus and gastroepiploic vein dilatation	Cystotomy	7 months	Survived
Serrano et al. [26]	USA	68	Female	Epigastric pain, hematemesis, and melena	Live segmental pancreas donation	Gastric varices	Laparoscopic adhesiolysis and splenectomy	1 month	Survived

Abbreviation: NA: not available.

**Table 3:** Literature review of case series regarding the prognosis of patients with left-sided portal hypertension.

References	Country	Study duration	Age (years)	Cases with left-sided portal hypertension	Primary pancreatic diseases	Intervention	Follow-up periods	Outcome
Sakorafas et al. [27]	USA	1976-1977	46 (19-74)	34	Chronic pancreatitis	Pancreatic surgery (*n* = 34)	NA	Died (*n* = 1)
Wang et al. [28]	China	1.2000-12.2009	43.5 ± 6.4	13	Chronic pancreatitis (*n* = 7) Pancreatic cancer (*n* = 3) Pancreatic cysts (*n* = 2) Neuroendocrine tumor (*n* = 1)	Surgical procedures (*n* = 13)	46 ± 7 months	Died (*n* = 3)
Liu et al. [16]	China	1.2001-12.2011	47 ± 8	21	Acute pancreatitis (*n* = 1) Chronic pancreatitis (*n* = 7) Pancreatic cancer (*n* = 12) Benign pancreatic tumor (*n* = 1)	Endoscopic variceal treatment (*n* = 5) Surgical procedures (*n* = 10) Splenic artery embolization (*n* = 6)	NA	Died (*n* = 13)
Zhang et al. [29]	China	1.1.1997-6.30.2012	46.3 ± 6.4	73	Pancreatic cancer	Radical operations (*n* = 35)	NA	Died (*n* = 25)
Rana et al. [30]	India	1.2012-11.2015	40.94 ± 8.43	18	Acute necrotizing pancreatitis	Transmural drainage (*n* = 18)	15.6 ± 12.2 weeks	All survived

Abbreviation: NA: not available.

## Data Availability

The data used to support the findings of this study are available from the corresponding author upon request.

## Abbreviations

LSPH: Left-sided portal hypertension
GI: Gastrointestinal
CT: Computed tomography
MR: Magnetic resonance
EGD: Esophagogastroduodenoscopy
PPI: Proton pump inhibitor.
